# Role of free fatty acids in endothelial dysfunction

**DOI:** 10.1186/s12929-017-0357-5

**Published:** 2017-07-27

**Authors:** Arijit Ghosh, Lei Gao, Abhimanyu Thakur, Parco M. Siu, Christopher W. K. Lai

**Affiliations:** 1 Department of Health Technology and Informatics, The Hong Kong Polytechnic University, HKSAR, China; 2 Department of Biomedical Sciences, City University of Hong Kong, HKSAR, China

**Keywords:** Free fatty acids, Endothelial dysfunction, Nitric oxide, Insulin resistance, Oxidative stress, Inflammation

## Abstract

Plasma free fatty acids levels are increased in subjects with obesity and type 2 diabetes, playing detrimental roles in the pathogenesis of atherosclerosis and cardiovascular diseases. Increasing evidence showing that dysfunction of the vascular endothelium, the inner lining of the blood vessels, is the key player in the pathogenesis of atherosclerosis. In this review, we aimed to summarize the roles and the underlying mechanisms using the evidence collected from clinical and experimental studies about free fatty acid-mediated endothelial dysfunction. Because of the multifaceted roles of plasma free fatty acids in mediating endothelial dysfunction, elevated free fatty acid level is now considered as an important link in the onset of endothelial dysfunction due to metabolic syndromes such as diabetes and obesity. Free fatty acid-mediated endothelial dysfunction involves several mechanisms including impaired insulin signaling and nitric oxide production, oxidative stress, inflammation and the activation of the renin-angiotensin system and apoptosis in the endothelial cells. Therefore, targeting the signaling pathways involved in free fatty acid-induced endothelial dysfunction could serve as a preventive approach to protect against the occurrence of endothelial dysfunction and the subsequent complications such as atherosclerosis.

## Background

Cardiovascular diseases (CVDs), particularly coronary heart disease (CHD), account for the major causes of mortality worldwide [1]. The presence of atherosclerosis is a very common characteristic in patients with CHD [1], and that endothelial dysfunction (ED) is suggested as one of the early events in the pathogenesis of atherosclerotic progression. The vascular endothelium is a tightly regulated organ that forms a vast interface between the blood and neighboring tissues, and is consisted of a monolayer of endothelial cells (ECs). It regulates a wide range of functions, including the maintenance of the balance between vasodilation and vasoconstriction, the maintenance of thrombosis and hemostasis, and other inflammatory responses in order to regulate the normal functioning in the circulatory system. However, various harmful stimuli such as oxidative stress and inflammation can alter the normal endothelium functioning and lead to the onset of ED. ED can be expressed as an imbalanced ratio of vasodilation to vasoconstriction induced by the endothelium, and such impairment in vasodilation is mainly due to the diminished release of nitric oxide (NO), the most important vasodilatory agent that is released by the ECs. Although impaired NO production is the main characteristic feature of ED, the production and/or utilization of other vasodilatory agents such as prostacyclin (PGI_2_) and bradykinin are also compromised in the context of ED.

Free fatty acids (FFAs), or non-esterified fatty acids (NEFAs), are well-known risk factors of CVDs [2], and are closely related to the events of metabolic syndromes (MetS), such as obesity and type 2 diabetes mellitus (T2DM) [3]. Recent studies have shown that FFAs not only are the major causes of insulin resistance [4, 5], but they are also responsible for inducing inflammatory events in the tissues targeted by insulin, such as ECs, liver and skeletal muscle [3, 6]. Therefore, elevated FFAs in blood are considered as an important link between insulin resistance, inflammation, obesity, T2DM and hypertension (HTN) [3]. The impairment of insulin-mediated glucose uptake is correlated to the circulating FFA levels, and such resistance to insulin might be due to FFA-mediated inactivation of phosphoinositide 3-kinase (PI3K) [7]. Interestingly, cancer-related up-regulation of FFAs was also reported in several types of cancer in many previous studies [8–11].

In addition, a growing body of evidence has suggested the important role of FFAs in mediating ED. Specifically, insulin resistance, oxidative stress, and inflammatory burdens account for the substantial causes of FFA-induced ED [12–14]. Generally, diabetes and other metabolic states could give rise to elevated FFAs, which in turn impose a direct effect on transcription factors that trigger inflammation and oxidative stress in the endothelium [15]. Furthermore, FFAs also facilitate apoptosis/necroptosis of the ECs [16–18], and mediate many deleterious effects on endothelial progenitor cells (EPCs) [19].

## Biology of the endothelium and endothelial dysfunction

### Distribution, structure, and functions of the endothelium

The endothelium is a composition of a monolayer of ECs which lines on the inner surface of the vascular lumen between flowing blood and vascular smooth muscle cells (VSMCs) [20]. The endothelium has a thickness of ≤1 μm and covers a surface area of 4000 m^2^ throughout the whole circulation [21]. ECs are multifunctional; they are responsible for a wide range of vital functions, including the maintenance of vascular tone, blood fluidity and permeability [21, 22]. The endothelium is also responsible for the regulation of inflammatory responses and signals for the regulation of hemostasis/thrombosis, fibrinolysis and angiogenesis [20, 21, 23]. Thus, impairment of the endothelial functions is suggested to play deleterious roles in the development of several diseases, including inflammatory angiitis syndrome, thrombotic embolism, disseminated intravascular coagulation (DIC) disorder, and neovascularization, tumor progression and diabetic retinopathy [23].

ECs regulate various pathophysiological properties by synthesizing and secreting various molecules relative of the blood and/or to the extracellular matrix [24]. For example, endothelium-secreted molecules such as angiotensin II (Ang II), endothelin-1 (ET-1), thromboxane A2 (TXA2), and prostacyclin H2 participate in vasoconstriction, whereas, molecules such as NO, bradykinin, and hyperpolarizing factor contribute to vasodilation that helps maintaining a balance between the vasoconstriction and vasodilation [20]. The fine balance between these secreted molecules is critical for a proper functioning of the endothelium, and an imbalance of these molecules may contribute to failure in vascular auto-regulation, and influence the structural and functional integrity of the circulation [24].

### Importance and regulation of nitric oxide (NO) in endothelial function

After the discovery of prostacyclin (or prostaglandin I2, PGI_2_) and endothelium derived relaxing factor (EDRF), NO was identified as the third endothelium regulator for vascular auto-regulation. In the endothelium, NO is synthesized from L-arginine, a semi-essential amino acid, by the endothelial nitric oxide synthase (eNOS), and L-citrulline is the by-product of this pathway [25]. The biosynthesis of NO also requires many co-factors, such as nicotinamide adenine dinucleotide phosphate (NADPH), flavin mononucleotide (FMN), flavin adenine dinucleotide (FAD), tetrahydrobiopterin (BH4), and calmodulin [25]. Following its synthesis, NO diffuses across the EC membranes and enters the VSMCs where it activates guanylate cyclase (GC). Activation of GC further gives rise to the intracellular cyclic guanosine-3′,5-monophosphate (cGMP), which, as a second messenger, mediates the biological actions of NO, including the control of vascular tone and platelet aggregation [25–27]. Shear stress is a crucial factor for the activation of eNOS under physiological circumstances; other signaling molecules such as bradykinin, adenosine, vascular endothelial growth factor (VEGF), and serotonin can also lead to the activation of eNOS [28].

### Endothelial dysfunction: Pathogenesis and mechanisms

ED can be described as diminished production and/or availability of NO, and an imbalance between the endothelium-derived vasodilators and vasoconstrictors. It is an event that accounts for the risk of CVDs and precedes the development of atherosclerosis [20, 29]. Among the relevant mechanisms of ED pathogenesis, proposed in previous studies, oxidative stress and inflammation account for the majority of them [12, 13, 29–31]. In particular, the inactivation of NO by oxidative enzyme systems such as NADPH oxidase, xanthine oxidase (XO), cyclooxygenases (COX), lipoxygenases (LOX), myeloperoxidases (MPO), cytochrome P450 monooxygenase, uncoupled NOS, and peroxidases is one of the critical mechanisms that leads to ED through an elevated level of superoxide anion (O_2_^−^) [31–33]. NADPH oxidase acts as an important source of O_2_^−^ that gives rise to vascular oxidative stress [31, 32], and approaches that could lower NADPH oxidase might have reversing effects on ED [14].

Also, eNOS uncoupling is another mechanism of ED that leads to increased O_2_^−^ synthesis instead of NO synthesis, in a scenario where BH4 is inadequate; restoration of BH4 can reduce uncoupled eNOS and reverse oxidative stress-induced ED [31, 34, 35]. Oxidative stress-induced ED is a major concern observed in patients with CVDs and MetS [30, 33, 36]. Many studies have suggested a role of inflammation in ED that underlies the pathogenesis of CVDs, obesity and T2DM. Elevated levels of pro-inflammatory cytokines such as tumor necrosis factor-alpha (TNF-α), interleukin-1beta (IL-1β), interleukin-6 (IL-6), and interferon gamma (IFN-γ) were observed in age-related ED, both in rodents and humans [37, 38], mainly via the activation of the NF-κB (nuclear factor-kappa B) pathway [37, 39, 40]. NF-κB, with the association of IκK, controls the global pro-inflammatory response in ECs [41], and acts as an effective “transducer for feed forward activation” of oxidative stress and inflammatory signaling, subsequently leading to ED by means of increased NADPH oxidase-mediated ROS upregulation, and upregulation of the pro-inflammatory cytokines such as IL-6 and TNF-α [42, 43]. Therefore, NF-κB is considered as a potential therapeutic target for the control and prevention of arterial aging and atherosclerosis [41, 42].

In addition, ED can also be induced by many factors such as dietary intake, drugs, and aging. For example, high-fat diet (HFD) induces a downregulation of the endothelial AMPK-PI3K-Akt-eNOS pathway which in turn causes ED; such downregulation of the AMPK-PI3K-Akt-eNOS pathway correlates with increased plasma levels of FFAs and TG, and an impaired glucose utilization [44]. Besides, uric acid (UA), which is a final product derived from purine metabolism pathway, can induce ED via the high mobility group box chromosomal protein 1/receptor for advanced glycation end products (HMGB1/RAGE axis). UA inhibits eNOS expression and subsequent NO production in human umbilical vein ECs (HUVECs), by increasing intracellular HMGB1 expression and extracellular secretion of the protein. UA also upregulates RAGE expression and other inflammatory cytokines, possibly via the activation of NF-κB transcription factor [45]. A role of HMGB1 in inducement of ED, triggered by FFAs, has also been shown in a previous study [46]*,* which will be further discussed later in *Section 4.3*; other mechanisms by which FFAs can mediate ED have been discussed in *Section 4*. Drug-induced generation of ROS and inflammation also play a major role in the onset of ED. Several classes of drugs, including anti-cancer drugs, immunosuppressive drugs, anti-retroviral drugs, and others, have been known to induce ED. For example, doxorubicin (DOX) induces ED in a VEGF-dependent manner, and targeting VEGF rescues DOX-induced ED [47, 48]. Alteration in endothelial markers such as PGH2, TXA2, NO, ET-1, thrombomodulin and von Willebrand factor (vWF) are seen as aging progresses. Notably, NO production is reduced in elderly people which has also been seen in aged animals through a downregulation of eNOS. On the other hand, ET-1 expression is also increased with aging; ET-1 inhibits acetylcholine (ACh)-dependent platelet inhibition in the endothelium and is a promoter of vWF expression. vWF is negative regulator of NO and a well-known marker of ED [49]. More recently, Medin, a common amyloidogenic protein in humans, especially in older individuals, has been shown to induce ED. In ex vivo human adipose and leptomeningeal arterioles, the protein could decrease NO production, increase peroxynitrite, and superoxide production and enhance the expression of the proinflammatory markers such as IL-6 and IL-8 as well as the NF-κB transcription factor. These effects were shown to be mediated through RAGE and inhibition of RAGE by its specific inhibitor, FPS-ZM1, could reverse all such changes [50].

Several compounds with therapeutic value against ED, in different diseased states, have been reported in the recent years. Rosuvastatin, a lipid-lowering statin, has been shown to improve endothelial function in patients with inflammatory joint diseases, systemic sclerosis and chronic heart failure (CHF) [51–53]. Particularly, in the CHF patients, the drug improved the flow-mediated dilation (FMD) by inducing antioxidant effects, neovascularization and Akt phosphorylation [53]. Protective roles of pitavastatin in obesity-related ED [54], by increasing FMD and lowering triglycerides, and gliclazide in T2DM-related ED [55], by increasing FMD and EPCs, have also been reported. Pioglitazone, a peroxisome proliferator-activated receptor gamma or PPARγ agonist, was shown to prevent ischemia-induced ED in healthy subjects [56], and improve endothelial and adipose tissue dysfunction in pre-diabetic patients with coronary artery disease (CAD) [57]. In the CAD patients, the drug could improve FMD, reduce TNF-α and other inflammatory markers, and improve insulin sensitivity [57]. A synergistic protective effect of pioglitazone with quercetin, a naturally occurring plant polyphenol, on ED has also been shown on isolated rat aorta with the characteristics of T2DM [58]. A detailed meta-analysis on the effects of thiazolidinediones on the FMD has been reported elsewhere [59]. In a more recent study, it has been shown that the drug could protect EPCs by upregulating long non-coding RNA maternally expressed 3 (lncRNA MEG3) in MetS patients [60]. However, it should be noted that pioglitazone can induce heart failure in patients with underlying heart disease [61]. Efficacy of dipeptidyl peptidase IV (DPP-IV) inhibitors against ED has also been reported [62]. Efficacy of more drugs with multifaceted mechanisms in different diseased states with ED has been reviewed elsewhere [63]. Butyrate, a 4-carbon FA, when given orally in ApoE^−/−^ mice, can lessen atherosclerotic development by reducing ROS load in ECs and macrophage migration and activation at the site of lesion; such reduction of ROS by butyrate is mediated through downregulation of the oxidative enzymes such as NADPH oxidase and iNOS in atherosclerotic lesions [64].

In addition, protective roles of many natural products, such as Traditional Chinese Medicines (TCM), have been reported to improve endothelial function. Tongxinluo, a TCM against CVDs, mediates protective role against ED in rats by means of lowering ET-1, and increasing NO [65]. Danshensu, another TCM, which is a water-extractable component of the medicinal herb *Salvia miltiorrhiza*, protects the endothelium in rats with hyperhomocysteinemia by modulating the abnormality in the parameters such as NO, ET-1, and other inflammatory markers induced by hyperhomocysteinemia [66]; homocysteine is a byproduct of numerous biological processes in the human body and, when elevated, it may be associated with severe atherosclerosis and thrombotic occlusions [67]. Some other types of TCM formulations with potential effects on the endothelium have been described elsewhere [68]. Other compounds having potential effects on endothelial function, which can be disturbed by FFAs, have been discussed in the later parts.

## FFAs and their role in diseases

### FFAs and their receptors

Fatty acids (FAs) are carboxylic acids with long aliphatic chains containing a methyl group at one end, while a carboxylic group at the other end. Depending on the presence of double bonds, they are classified into saturated fatty acids or SFAs with no double bonds, monounsaturated fatty acids or MUFAs with only one double bond and polyunsaturated fatty acids or PUFAs with at least two double bonds [69]. SFAs are primarily derived from animal and dairy products, coconut and palm oils, whereas unsaturated FAs (UFAs) such as MUFAs and PUFAs are found in olive oil, nuts and in some fatty fishes [69].

FAs, depending upon their amino acid chain lengths, can also be divided into three types: short-, medium-, and long-chain FAs (SCFAs, MCFAs, and LCFAs, respectively) [70]. SCFAs contain no more than 6 carbons, while MCFAs contain 6–12 carbons, and LCFAs have more than 12 carbons. Saturated FAs such as acetic acid (C2:0), propionic acid (C3:0), butyric acid (C4:0), valeric acid (C5:0) and caproic acid (C6:0) are examples of SCFAs, while caprylate (C8:0), capric acid (C10:0) and lauric acid (C12:0) are MCFAs; other SFAs such as myristic acid (C14:0), palmitic acid or PA (C16:0) and stearic acid or SA (C18:0), and all types of unsaturated FAs, including MUFAs and PUFAs, fall under the category of LCFAs [71].

The FFA sensing receptors (FFARs) belong to the G-protein or guanine nucleotide-binding protein coupled receptors (GPCRs) family [72]. Several GPCRs, including GPR40 (FFAR1), GPR43 (FFAR2), GPR41 (FFAR3) and GPR120 (FFAR4), have been found to be activated by extracellular FFAs [70, 73]. While SCFAs bind to FFAR2 and FFAR3, the other two subtypes, MCFAs and LCFAs, bind to FFAR1 and FFAR4. The endogenous ligand potency (EC_50_) of FFA2 and FFA3 for SCFAs is 0.1–1.0 mM, while that of FFA1 and FFA4 for LCFAs is 1.0–30 μM [73]. Detailed physiological and pathophysiological roles of these receptors have been described elsewhere [72, 73].

### FFAs and CVDs

SFAs are largely responsible for CVDs, while UFAs are unlikely to cause CVDs, and rather UFAs are mostly found to be protective against CVDs. SFAs increase low-density lipoprotein (LDL), which is a major risk factor for CVDs [74]. Studies suggest that LCFAs bear greater risk for CHD than that by SCFAs or MCFAs [74]. The most common LCFAs occurring in western diets are myristic acid (14:0), PA (16:0) and SA (18:0). Previous plasma metabolomic studies have confirmed PA as a strong contributing factor to the development of atherosclerosis [75]. Several recent in vivo and in vitro studies have revealed the mechanisms by which PA contributes the pathogenesis of CVDs. A very recent study has shown that PA is a promoter of inflammatory responses and cellular senescence in cardiac fibroblasts which it mediates via the activation of toll-like receptor 4 (TLR4) and NLRP3 inflammasome, increasing mitochondrial ROS load and mitochondrial dysfunction, and functionality loss of the cardiac fibroblasts [76]. Another study has shown that PA could mediate apoptosis of the VSMCs by inducing the TLR4 pathway and ROS generation [77]. The specific roles and mechanisms of FFAs, in particular through mediation of ED, in the development of CVDs have been discussed in the later parts of this article *(Section 4)*.

### FFAs and insulin resistance: Role of oxidative stress and inflammation

FAs possess different physiological roles - structurally they contribute to the constituents of the membrane lipids, including phospholipids and glycolipids, whereas, functionally they are important as fuel molecules [78]. Although they are important sources of energy, particularly during a fasting condition, abnormalities in FA metabolism may contribute to the pathogenesis of MetS [78], and may bear risks for developing atherosclerosis [79]. In obesity, high levels of plasma FFAs are seen because of several reasons, such as release of more FFAs by enlarged adipose tissue mass and that the FFAs clearance may also be compromised in obesity [3, 80]. In turn, higher levels of FFAs inhibit the anti-lipolytic action of insulin, which further increases the rate of FFAs release into the circulation [81]. Clinical studies have shown that elevated level of FFAs leads to an insulin-resistant state, and that lowering of FFAs can be beneficial to insulin-stimulated glucose uptake [3]. Several mechanisms underlie FFA-induced insulin resistance such as intracellular accumulation of triglycerides (TG) and diacylglycerol (DAG), activation of serine/threonine kinases, reduced tyrosine phosphorylation of the insulin receptor substrate 1/2 (IRS 1/2) and impairment of the IRS/phosphoinositol 3 kinase (PI3K) pathway, involved in insulin signaling [3, 80]. By inducing an insulin-resistant state in all major insulin target organs, including the ECs, liver, and skeletal muscles [6, 80], FFAs contribute to the progression of T2DM, HTN, dyslipidemia and nonalcoholic fatty liver disease (NAFLD) [80]. T2DM, which is manifested by a chronic insulin-resistant state with progressive decline in functional status of β-cells, is often associated with hypertriglyceridemia or increased plasma concentrations FFAs [78].

FFAs are significant sources of reactive oxygen species (ROS), which lead to the event of oxidative stress. Not only in vascular cells [82], but also in other cells types, such as hepatocytes [83], and immune cells [84], FFAs lead to the generation of ROS, mainly through the activation of NADPH oxidase via protein kinase C (PKC) [82, 84]. A role of PKC is also seen in FFA-induced inflammation; FFA-mediated inflammation also relates to the IKK/NF-κB inflammatory signaling and leads to the activation of TNF-α, IL1-β, and IL-6, and increased plasma levels of the monocyte chemotactic protein-1 (MCP-1) [80]. All these inflammatory components play a role in chronic inflammation that might cause insulin resistance in the ECs [85, 86].

Interestingly, FFAs are also modulators of the NLRP3 inflammasome in the context of T2DM and obesity [87]. Inflammasomes act as both innate immune system receptors and sensors, and regulate a number of activities such as the activation of caspase-1 and inducement of inflammation; NLRP3 is the best characterized inflammasome [87] that can correlate to a number of human diseases, including atherosclerosis, MetS and neurodegenerative diseases [88]. Palmitate or PA has been shown to activate NLRP3, which in turn can enhance ROS generation in macrophages and subsequently weaken the AMPK signaling, which is a negative regulator ROS generation and inflammation [89]. Using a mouse liver cell line, it was also shown that palmitate is an inducer of IL-1β, which could suppress the insulin-induced Akt phosphorylation, suggesting the development of insulin resistance by FFA through the mediation of ROS and inflammatory signaling via the NLPR3 inflammasome.

## Role of FFAs in inducing ED: Evidence from clinical and experimental studies

### FFAs induce ED via downregulation of the AMPK/PI3K/Akt/eNOS signaling pathway

Insulin is a key mediator of NO-mediated vasodilation [90], and 5′ adenosine monophosphate-activated protein kinase (AMPK) is important in insulin signaling that serves as a target for against insulin resistance in MetS and related diseases [91, 92]. Moreover, AMPK plays a role in FA oxidation [93]. AMPK, which is a fuel-sensing enzyme, is activated by an increased AMP/ATP ratio following a number of stimuli such as exercise or its pharmacological activators, and can further phosphorylate and inactivate Acetyl-CoA carboxylases (ACC), which catalyzes the synthesis of malonyl-CoA and is important for FAs synthesis, a mechanism by which AMPK increases the oxidation of FAs [86, 93], thus serving as a target in MetS-related elevation in FAs. Not only so, but also that AMPK can modulate both oxidative stress and NF-κB-mediated inflammatory signaling [86]. FFAs downregulate insulin-mediated production of NO and peripheral blood flow via two mechanisms: 1) by reducing tyrosine phosphorylation IRS-1/2 and 2) by inhibiting the PI3K/Akt pathway, which not only controls insulin-stimulated uptake of glucose but also regulates eNOS → NO production in the ECs [80]. One study showed that increased level of FFAs in the bloodstream impairs endothelium-dependent vasodilation while the endothelium-independent vasodilation remains unaffected [94], suggesting FFAs have specific inhibitory roles on production of NO in the ECs. Another study reported that infusion of FFA in insulin-sensitive human subjects leads to a significant reduction in NO synthase flux and an impaired shear stress-induced production of NO [95]. More recently, effects of FFAs have been shown on eNOS activity by inhibiting eNOS mRNA expression in rat aortic ECs and modulating eNOS activity through possible increases in oxidative stress and inflammatory burdens [96]; a similar downregulation in insulin-mediated eNOS activity by FFAs was shown through upregulation of PTEN (phosphatase and tensin homolog) and simultaneous inhibition of Akt kinase [97]. The AMPK/PI3K/Akt/eNOS pathway relative of ED has been extensively reported, which is diminished with the increasing level of FFAs, induced by HFD [98]. Detrimental roles of the SFAs such as PA and SA were shown in porcine aortic ECs (PAECs) through the downregulation of eNOS [99]. Interestingly, even though PUFAs have a protective role against ED [100], their free circulating form could also mediate a negative action on the endothelium by decreasing NO availability and increasing ET-1 [101]. Storniolo and colleagues reported that free forms of linoleic acid (LA), which is an ω-6 PUFA, negatively regulate eNOS phosphorylation, and consequently affect the level of intracellular NO availability in ECV304 cells [102].

Protective role of several dietary constituents has been shown in FFA-induced ED via the AMPK/PI3K/Akt/eNOS pathway. For example, eicosapentaenoic acid (EPA), which is an ω-3 or n-3 PUFA, has a protective role against PA-induced ED which is mediated via activation of the AMPK/eNOS pathway; EPA also mediated its inhibitory effect on the PA-induced apoptosis of ECs and activation of apoptosis-related proteins, such as Caspase-3, p53 and Bax [18] (Table 1). Decreases in NO synthesis and increase in the level of ET-1 can be reversed by treatment with the olive oil polyphenolic compounds [102]. There are many clinical studies reporting the protective effects of EPA or olive oil constituents such as oleic acid (OA) on FMD and other endothelial markers [103–105], which, however, did not focus on their effects on FFA-induced ED. Although the studies by Lee et al. [18] and Storniolo et al. [102] have strong implications that EPA and olive oil polyphenols can be beneficial in FFA-induced ED, these studies were limited to the use of in vitro models only; further animal or even human studies should be carried out to support the effects of these compounds on FFA-induced ED. A role of the mitochondria-related AMPK/eNOS pathway has also been shown to alleviate ED and atherosclerosis in mice fed with HFD [106]. Salidroside (SAL), an isolated phenylpropanoid glycoside from the medicinal plant *Rhodiolarosea*, mitigates ED and atherosclerosis by activating the mitochondrial AMPK/PI3K/Akt/eNOS pathway and promoting NO production [106]. Moreover, L-carnitine, an important factor for FA transport/oxidation in the mitochondria, attenuates FFA-induced obesity-related ED in human subjects [107] (Table 1).

**Table 1:** Drugs/dietary constituents with beneficial effects against FFAs-induced ED. This table shows the reported dietary nutrients/drugs that have been shown to be effective against FFAs-induced ED, and the potential mechanisms highlighted in these studies. Two differently-colored texts in the table have been used to highlight different studies on the same drug or the use of more than one study model used within the same article

Drug/dietary constituent	Effects/relevant mechanisms	Nature of the study
ω-3 PUFAs (EPA)	AMPK/eNOS pathway ↑	In vitro study on primary HUVECs [18]
	iNOS ↓	
	EC apoptosis, Caspase-3,	
	p53/MAPK, Bax ↓	
	NADPH oxidase/ROS ↓	
	NF-κB activation ↓	
*Astragalus membranaceus*	NO ↑	Ex vivo study on rat aortic rings [134, 141]
	Endothelium-dependent	
	vasodilation ↑	
	NF-κB ↓	
Cyanidin-3-O-glucoside	Oxidative stress ↓	In vitro study on primary HUVECs [131]
	NF-κB activation and adhesion	
	molecules ↓	
	Nrf2/EpRE pathway ↑	
Dihydropyridine calcium channel blockers (Nifedipine and amlodipine)	Forearm blood flow responses to	Clinical trial [128]
	ACh ↑	
	Leucocyte activation ↓	
	Oxidative stress ↓	In vitro monocytic cells [128]
	NF-κB ↓	
	TNF-α, IL-6 ↓	In vitro study on HUVECs [131]
	IKKβ/NF-κβ phosphorylation ↓	
	IRS-1 phosphorylation ↓	
	NO production ↑	
L-carnitine	Endothelium-dependent leg blood flow ↑	Clinical trial [107]
Losartan	Vasodilation ↑	Clinical study [139]
	eNOS activity ↑	
	IRS-1 phosphorylation ↓	Study on rats [139]
Olive oil polyphenols	eNOS activity ↑	In vitro study on ECV304 cells [102]
	ET-1 ↓	
Perindopril	Vasodilation ↑	Clinical study [141]
Salidroside	eNOS activation, NO production ↑	In vivo study on HFD-fed ApoE^−/−^mice [106]
	AMPK/PI3K/Akt/eNOS pathway,	
	Cellular AMP/ATP ratio ↑	
	Atherosclerotic lesion ↓	
Withaferin A	ROS, TNF-α, IL-6 ↓	In vitro study on primary HUVECs [129]
	IKKβ/NF-κβ phosphorylation ↓	
	IRS-1 phosphorylation ↓	
	PI3K signaling ↑	
	ET-1, PAI-1 ↓	Ex vivo study on rat aortic rings [129]
	Endothelium-mediated vasodilation ↑	

### FFAs-induced ED through disruption of calcium signaling-mediated NO production/release

**Fig. 1:**
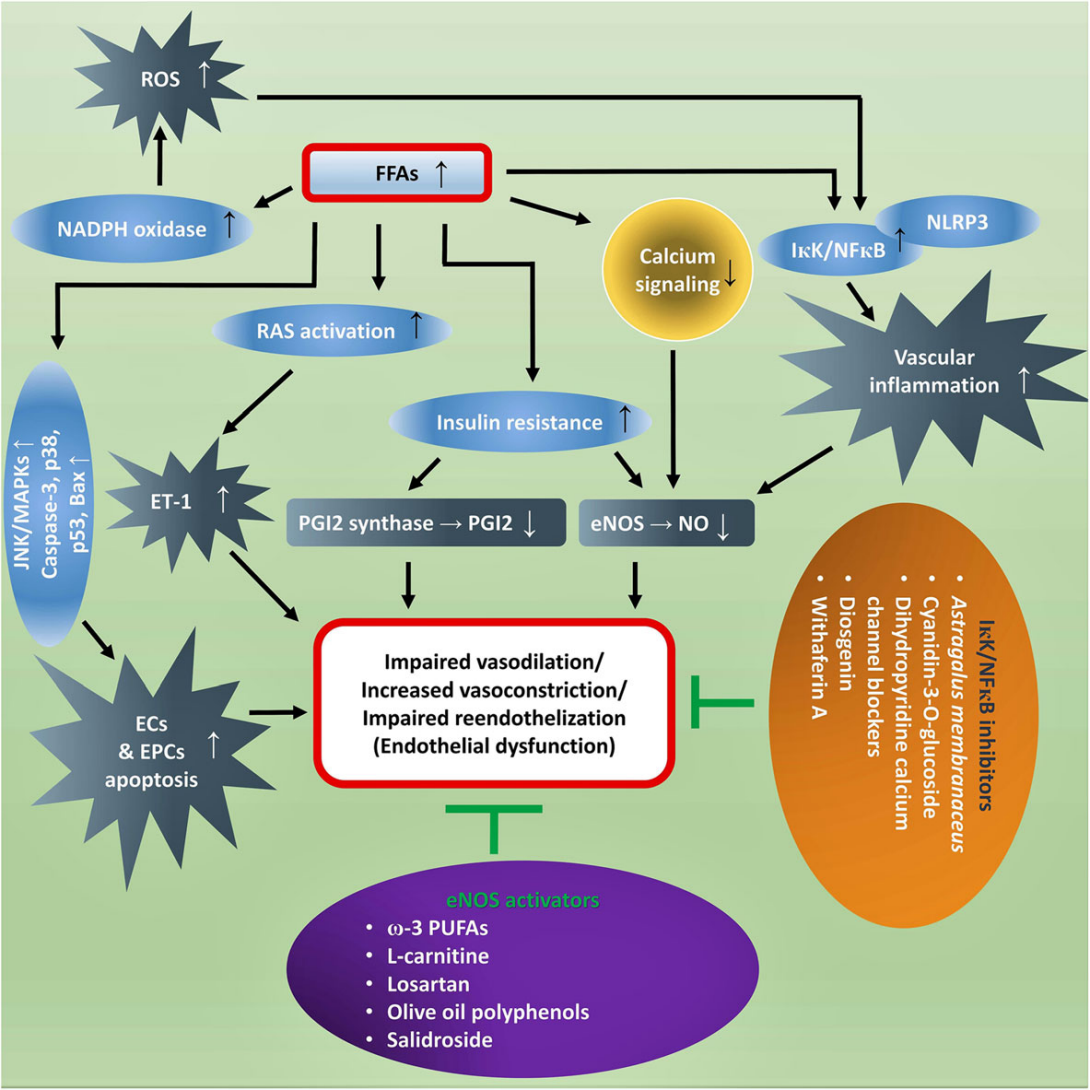
Potential mechanisms by which FFAs induce ED. FFAs mediate ED by means of several mechanisms, which might have direct/indirect effects on NO production. For example, FFAs can mediate oxidative stress and inflammation in the endothelium which can affect insulin signaling and contribute to dysregulated NO production. Activation of the RAS by FFAs can elevate the level of ET-1, which can lead to vasoconstriction. Furthermore, FFAs can also activate the apoptotic pathways which can induces apoptosis in ECs and EPCs. Several dietary/therapeutic agents can be beneficial against FFA-induced ED through activation of the eNOS or via the inhibition of NF-κB-mediated inflammatory signaling (has been listed in Table 1)

Calcium signaling plays a crucial role in endothelial function by facilitating the release of NO through activation of eNOS, which is a calcium-dependent enzyme [108]. Several receptor-operated agonists, such as adenosine 5′-triphosphate (ATP) and bradykinin facilitate breakdown of phosphatidylinositol, leading to an increase in the intracellular free calcium concentration [109]. Intracellular calcium is important for mechanosensitivity responses of ECs [110], and that shear stress-mediated release of NO largely depends on increases in cytosolic calcium levels [111, 112]. Free forms of UFAs such as oleic acid (OA) and linoleic acid (LA) diminish ATP-induced mobilization and influx of intracellular calcium in bovine aortic endothelial cells (BAECs) culture, and thus impair production/release of NO [113]. Later on, another study confirmed the deleterious effects of FFAs on endothelial calcium signaling and subsequent eNOS activity that led to diminished NO production [114] (Fig. 1).

### Role of the FFAs in mediation of oxidative stress and inflammatory signaling in the endothelium

Oxidative stress-related ED is not only a critical mechanism that leads to CVDs [30], but it is also a major contributor to the pathogenesis of MetS [3]. A dose-dependent increase of ROS is seen in monocytes exposed to FFAs which leads to adhesion of the monocytes to the ECs [115] - a critical mechanism in the development of atherosclerosis at the earlier stages [116]. Interestingly, FFAs-induced increases in CV risk factors, characterized by elevated levels of endothelial markers, is seen in healthy subjects, a possible mechanism in the development of the CVDs [117]. Besides, HFD, which is a large source of FFAs, also induces oxidative stress in endothelium [118, 119]. Chinen and colleagues showed that FFA induces overexpression of NADPH and mediates oxidative stress in rats with characteristics of both obesity and T2DM [120]. Events of ER stress induced by FFAs have also been shown in ECs isolated from healthy human subjects [121]. The study has shown that intralipid infusion in healthy subjects could lead to 4.2-fold increases in the level of FFAs which was associated with a reduction of the hyperemic response in the leg without a change in FMD of the brachial artery. They also have revealed that mRNA levels of the genes ATF6 and IRE1, which are responsible for early adaptive responses to ER stress, had also been elevated in ECs; however, such changes were only adaptive rather than apoptotic.

FFAs may contribute to inflammatory states that lead to enhance endothelial permeability [122]. One of the major pathways leading to FFAs-induced ED is the activation of NF-κB, reported in many studies [123]. Intake of trans fatty acids (TFAs), consumed through foods made from partially hydrogenated vegetable oils, can activate the NF-κB pathway, leading to increased endothelial superoxide production and reduced NO production [124]. The NF-κB pathway is a major player mediating the deleterious effects of SFAs on human coronary artery ECs [125]. Surprisingly, FFAs of PUFA such as LA also may play a role in inducing inflammatory responses by increasing the levels of TNF-α, MCP-1, vascular cell adhesion molecule 1 (VCAM-1), and intercellular adhesion molecule 1 (ICAM-1) through the activation of NF-κB and activator protein 1 (AP-1) [126], and affect the release of NO [113]. Interestingly, a study has shown that IKK-β, which is an activator of NF-κB, can also diminish NO production [127] (Fig. 1). Moreover, a role of the FFAs in inducement of the NLRP3 inflammasome has been shown that could lead to an increase in the endothelial permeability [122]. In microvascular endothelial cells (MVECs), using palmitate, the authors have showed that it could activate the NLRP3 inflammasome with a resulting reduction in endothelial tight junction proteins - zonula occludens-1 and -2 (ZO-1 and ZO-2). Further exploring of the mechanisms, it had been found that FFAs mediated such effects by triggering the production of HMGB1 which might explain the early onset of endothelial injury during obesity.

Protective effects of several drugs and other dietary/natural agents through suppression of inflammation and oxidative stress in FFA-induced ED have been reported both in clinical and experimental studies. For example, dihydropyridine calcium channel blockers such as nifedipine and amlodipine possess preventive effects against FFAs-induced ED, leucocyte activation and oxidative stress as evidenced by studies in human subjects; by exploring the further mechanisms, through in vitro study, the authors showed a role of NF-κB in such mediation of FFAs-induced ED, and that the protective role of the drugs were through the suppression of NF-κB p65 phosphorylation [128]. A role of Withaferin A (WA), a steroidal lactone derived from *Acnistus arborescens* [129], against PA-induced insulin resistance and dysfunction of the endothelium has been shown, mediated through its anti-oxidant and anti-inflammatory properties [130]. Such inhibition of the inflammatory responses by WA was through suppression of IKKβ/NF-κβ phosphorylation and decreased release of proinflammatory cytokines, such as TNF-α and IL-6. Furthermore, insulin sensitivity in the ECs was shown to be improved via the PI3K signaling while inhibiting inflammation-stimulated IRS-1 serine phosphorylation. Similar inhibitory effects on IKKβ/NF-κβ phosphorylation, and TNF-α and IL-6 upregulation, induced by PA in HUVECs, were shown to be exerted by Diosgenin, a steroidal saponin extracted from *Dioscorea* [131]. Diosgenin, in the same study, also reportedly improved insulin signaling by modulating serine/tyrosine phosphorylation of IRS-1 and thereby inducing insulin-mediated NO production. Cyanidin-3-O-glucoside (C3G), an anthocyanin which is abundant in human diet, was reported to provide protection to the endothelium from PA-induced toxicity via suppression of NF-κB pathways and adhesion molecules [132]. Moreover, the compound could mediate nuclear localization of Nrf2 and thereby activating its related pathways to suppress oxidative stress and increase the expression of the cytoprotective genes in HUVECs. In another study, recently reported by the same authors, showed that the protective effects of C3G in HUVECs via the modulation of PA-induced ED (suppressed eNOS expression and NO release) was related to the PI3K/Akt axis and that C3G was a direct activator of Nrf2 [133]. Protective effect of *Astragalus membranaceus* on FFA-induced dysfunction in ECs in an anti-NF-κB manner has also been reported [134] (Table 1).

### Evidence of renin-angiotensin system activation by FFAs

The renin-angiotensin system (RAS) is a crucial regulator of the arterial blood pressure, and Ang II is known as a potent vasoconstrictor. EC membrane expresses the angiotensin converting enzyme (ACE), which is required for Ang II synthesis; Ang II causes vasoconstriction by stimulation of ET and depletion of NO, and inhibition of ACE is fruitful in boosting the NO pathway [135]. While Ang II gives rise to FFAs through downregulation of the FA oxidation pathway [136], FFAs, on the other hand, have also been shown to be activators of the RAS [137]. Mice lacking ACE (ACE^−/−^) show increased gene expression of enzymes related to lipolysis and FA oxidation [138], which might explain the interplay between RAS and generation of FFAs. Moreover, Ang II interferes with insulin signaling, mainly by affecting insulin-induced tyrosine phosphorylation of IRS-1 [139]. Activation of the RAS by Ang II or the activity of Ang II itself, has been implicated in the pathogenesis of ED [140, 141]. While leukocytes activation is deleterious for endothelial health, FFAs can activate leukocytes and contribute to the adhesion properties of leukocytes in an Ang II-dependent manner, leading to the onset of ED [140], and inhibition of the RAS is preventive against the FFA-induced ED in humans [141] (Fig. 1). In the latter study, Watanabe and colleagues showed that a single dose of either losartan, an Ang II receptor antagonist, or perindopril, an inhibitor of ACE, could completely prevent the FFAs-induced dysregulation of endothelium-dependent vasodilation, suggesting the blockade of RAS as an effective treatment for FFAs-induced ED. Interestingly, a more recent report studying the effect of losartan on Goto-Kakizaki (GK) rats, which mimic the symptoms of T2DM, showed that the drug could improve eNOS activity, possibly by modulating Ang II-mediated increase in phospho-IRS-1 [139].

### Effects of FFAs on endothelial progenitor cells (EPCs)

EPCs participate in endothelial recovery following arterial injury, and factors such as oxidative stress contribute to dysfunction and apoptosis of the EPCs [142]. Dysfunctional EPCs are thought to be key regulators in the pathogenesis of atherosclerosis and other CVDs [143]. PA, which is the most abundant type of FFAs in the circulating blood, contributes to apoptosis of EPCs which is facilitated via the p38 and JNK/MAPKs pathways [19] (Fig. 1). Another study also depicted a deleterious role of PA on EPCs in MetS patients via regulating lncRNA MEG3 [144]. MEG3 is required for human mesenchymal stem cells (hMSCs) differentiation into ECs [145]; however, some studies showed that MEG3 may also interfere with the proliferation and angiogenesis in VECs and its expression may correlate with cardiovascular aging [146]. These data raise a conflict of interest for which the pathophysiological roles of MEG3 needs to be studied with a deeper understanding in both EPCs and ECs.

## Perspectives

### Targeting the AMPK/PI3K/Akt/eNOS signaling pathways: Importance of insulin and other endogenous targets

The AMPK/PI3K/Akt/eNOS signaling pathways are important for NO synthesis, and disruption of this signaling in the endothelium induces ED [98]. In the endothelium, insulin induces production of NO via the IRS-1/IRS-2 signaling. IRS1 activates the PI3K/Akt, which, in turn, phosphorylates eNOS at Ser1177, catalyzing the conversion of L-arginine to L-citrulline and NO [147]. Moreover, insulin protects the endothelium by inhibiting Caspase-mediated ECs death and inducing antioxidant enzymes, such as heme oxygenase 1 (HO-1) through the PI3K/Akt pathway [147]. However, patients with obesity or T2DM show higher levels of FFAs that lead to insulin resistance [4], which further contributes to ED, a pivotal step in the initiation and progression of atherosclerosis [148]. Thus, mitigating insulin resistance through the upregulated AMPK/PI3K/Akt/eNOS pathway seems to be a crucial therapeutic opportunity to combat FFAs-induced ED and subsequent CVD events. Additionally, a study with direct infusion of insulin in the rats showed that hyperinsulinemia could mitigate FFA-induced ED in rat aortic rings [149]; however, the direct effect of insulin infusion still remains questionable as the article is in a non-English language with unclear mechanisms.

**Fig. 2:**
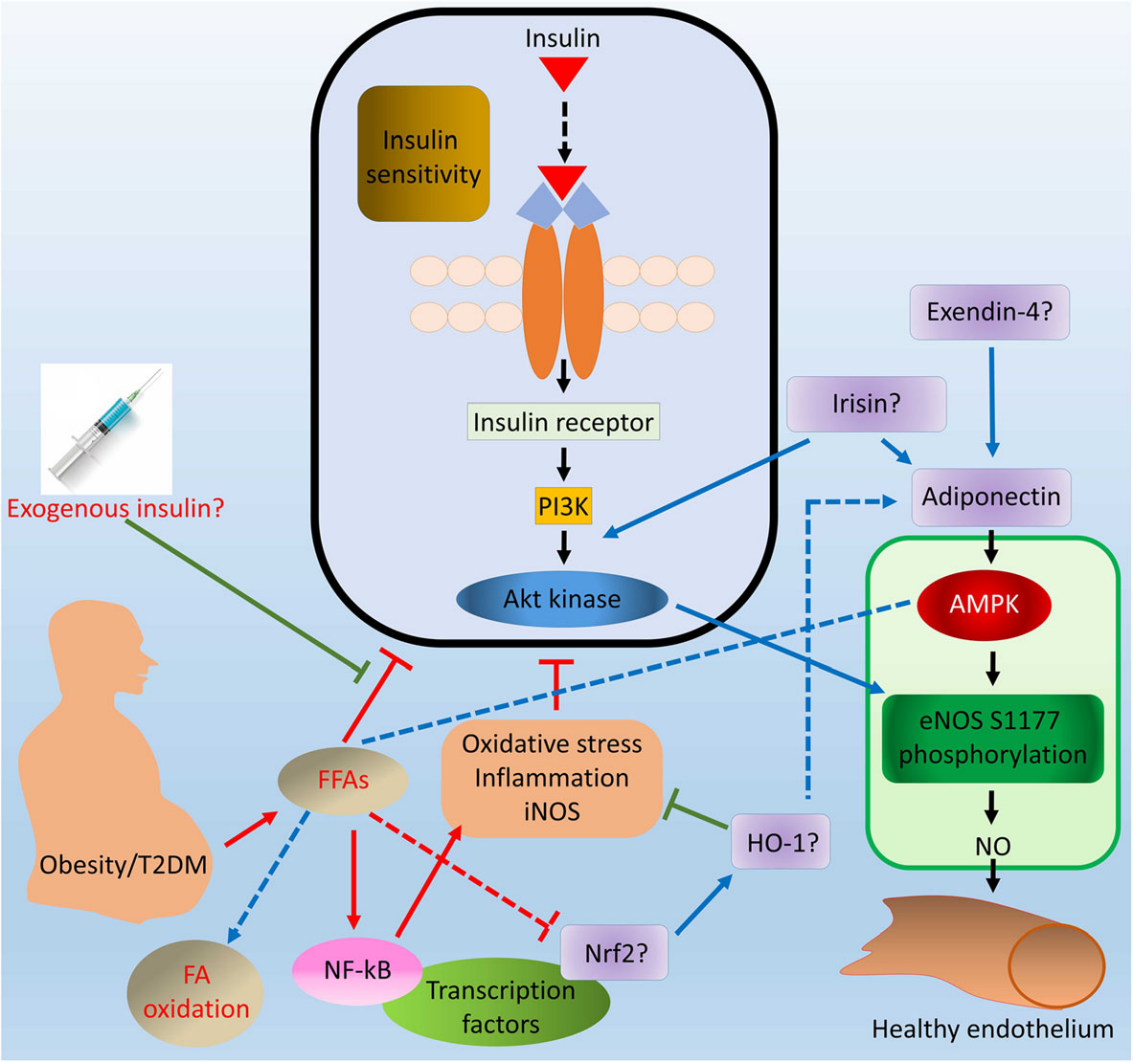
Possible targets against FFAs-induced ED. The level of FFAs is elevated in obesity/T2DM which contributes to ED. For a healthy endothelium, a balance in the relative pathways should be maintained. Here, we highlight some targets that may serve as important therapeutic avenue against FFAs-induced ED. These targets include Irisin and Exendin-4, which can increase eNOS activity by several means, but their role in ED, specifically induced by FFAs, should be studied. The Nrf2/HO-1 axis, which is a modulator of oxidative stress, might also have great impact to overcome FFAs-induced ED

Here, we propose that Exendin-4 and Irisin could be two novel targets against FFAs-induced ED. Exendin-4, also having its synthetic counterpart known as exenatide, a glucagon-like protein-1 (GLP-1) receptor agonist [150], increases insulin sensitivity via a PI3K-dependent mechanism [151]. Prevention of HFD-induced insulin resistance by Exendin-4 has been shown to be mediated through an increasing level of adiponectin [152]. It also mediates a direct improvement of the endothelial function via the cAMP or AMPK-eNOS pathways in isolated aortas from obese rats [153]. As FFAs have a role in downregulation of the AMPK-eNOS pathway, which is commonly observed in obesity, it can be suggested that Exendin-4 might regulate the activity of FFAs in obesity and thus improve obesity-related AMPK-eNOS pathway or endothelial function, which could be through the protective effects of Exendin-4 on FFA-induced apoptosis of the pancreatic β-cells [154, 155]. A role of Irisin in improving endothelial function via the AMPK-eNOS pathway is also noteworthy in obese mice [156] and it also has a protective role against ED and atherosclerosis in apolipoprotein E-Null (apoE(−/−)) diabetic mice [157]. A recent study reported that Irisin improves FA oxidation through the AMPK signaling pathway and glucose utilization in mouse model of T2DM [158]; in the same animal model, Irisin also reportedly inhibited hepatic gluconeogenesis via the PI3K/Akt pathway [159]. Moreover, a clinical study has suggested an antagonistic effect of Irisin on fatty acid binding protein 4 (FABP4), the FA binding protein, which is associated with an increased risk of obesity-related metabolic disorders and HTN [160]. These collective data suggest a possible protective role of Exendin-4 and Irisin on FFA-induced ED that would require further validation (Fig. 2).

### Nuclear factor erythroid 2-related factor 2 (Nrf2)/HO-1 pathway: A future therapeutic avenue against FFAs-induced ED?

In this part, we propose that the Nrf2/HO-1 pathway could be an important target pathway for the alleviation of FFA-induced ED. This pathway is an important regulator of oxidative stress, and its activation has been proved to be fruitful in many diseases through the modulation of oxidative stress, and inflammation [161–165]. Moreover, the pathway improves diet-induced cognitive deficits and fatty liver [166, 167]. Nrf2, which belongs to the subfamily of basic region leucine zipper (bZip) transcription factors, is responsible for cellular defense mechanisms against oxidative stress and is also crucial for suppression of signaling cascades relative of inflammation [168]. It plays a protective role on FFA-induced cardiotoxicity and ED [132, 169], possibly due to its direct effects on mitochondrial FA oxidation [170, 171]. On the other hand, HO-1, which also possesses anti-oxidative properties and is transcriptionally regulated by Nrf2 [172, 173], is suppressed during oxidative stress-induced EC injury [174], and inducing HO-1 in the mouse endothelium is favorable against T2DM-induced vascular injury, where HO-1 facilitates reendothelialization by increasing the number of EPCs through AMPK-mediated mechanism [175]. An anti-apoptotic role of HO-1, as well as its isoform HO-2, is found in glutamate-induced toxicity and oxidative stress in the cerebrovascular endothelium [176], where it plays a defensive role against disruption of the blood-brain barrier (BBB) and neurological deficits in stroke via the Nrf2 signaling [177]. Induction of HO-1 has been shown to improve FFA-induced ED in rat aorta by activating the AMPK/PI3K/eNOS pathway [178]. This study also showed that adiponectin, which is an anti-inflammatory protein, is induced by HO-1. Interestingly, adiponectin, which mediates its protective role in cAMP-dependent alleviation of FFA-induced ED [179], is endogenously regulated by HO-1 in obese and diabetic animal models [180, 181], suggesting an important role of HO-1 in regulation of FFA-induced endothelial toxicity through multifaceted mechanisms. Looking at the several important roles of the Nrf2/HO-1 pathway, such as suppression of oxidative stress and inflammation, improvement of insulin resistance [166], inhibition of phosphorylation of PI3K/Akt [165, 167], inhibition of NF-κB [165], as well as their individual suppressive role (independent of each other) on FFA-induced ED [132, 178], it could be endorsed that the pathway could be an avenue for future therapy for FFA-related ED (Fig. 2).

## Conclusions

ED is an early event in atherosclerosis and other CVDs. FFAs, which are elevated in blood due to metabolic defects under different diseased states, such as obesity, and T2DM, contribute to ED and subsequent events of CVDs, by means of several mechanisms such as decreased insulin signaling and NO production, impaired endothelium/insulin-dependent vasodilation, and increased oxidative stress and inflammation. Thus, early intervention of FFA-induced ED could be beneficial against the CVDs related to ED. Choosing the right lifestyle, such as cutting HFD and eating foods rich in ω-3 or ω-6 FAs or other dietary components that have proven protective role on the endothelium, could be a preventive approach against ED. However, as it is proven that the circulating forms of ω-3 or ω-6 FAs could also sometimes contribute to oxidative stress and inflammation and thus cause ED, it is not completely feasible to conclude the idea of taking more ω-3 or ω-6 FA-rich foods. Therefore, it would require a better understanding of this field and identify some better, possible targets that could be used to develop better therapeutic approaches to intervene the early events of ED-related health conditions and pave the way for a better living.

## Abbreviations

AMPK: 5′ adenosine monophosphate-activated protein kinase
CVD: Cardiovascular disease
ED: Endothelial dysfunction
EPCs: Endothelial progenitor cells
FA: Fatty acid
FFA: Free fatty acid
HO-1: Heme oxygenase 1
MUFA: Monounsaturated fatty acid
MVECs: Microvascular endothelial cells
NADPH: Nicotinamide adenine dinucleotide phosphate
NF-κB: Nuclear factor kappa B
NO: Nitric oxide
NOS: Nitric oxide synthase
Nrf2: Nuclear factor erythroid 2-related factor 2
PA: Palmitic acid
PGI_2_: Prostacyclin
PI3K: Phosphoinositide 3-kinase
PUFA: Polyunsaturated fatty acid
ROS: Reactive oxidative stress
SFAs: Saturated fatty acids
T2DM: type 2 diabetes mellitus
VSMCs: Vascular smooth muscle cells
